# Isolation and identification of group A rotaviruses among neonatal diarrheic calves, Morocco

**DOI:** 10.1186/s13104-016-2065-8

**Published:** 2016-05-05

**Authors:** Imane Ennima, Ghizlane Sebbar, Bachir Harif, Saaid Amzazi, Chafiqa Loutfi, Nadia Touil

**Affiliations:** Laboratory of Biochemistry and Immunology, Department of Biology, Faculty of Sciences, 4 Avenue Ibn Batouta, B.P. 1014 RP, Rabat, Morocco; Société de Productions des Produits Biologiques & Vétérinaires, Avenue Hassan II, 10051 Rabat, Morocco; Equipe de Recherche en Virologie Moléculaire et Onco-Biologie, Faculté de Médecine et de Pharmacie, Université Mohamed V, Av. Mohamed Belarbi El Alaoui, Rabat, Morocco

**Keywords:** Bovine rotavirus, Isolation, G6 P[5], G10 P[14], Morocco

## Abstract

**Background:**

Group A rotaviruses (RVA) are the main cause of neonatal calve diarrhea (NCD) in Morocco. In this study, we isolated RVA strains from NCD clinical samples in order to support RVA disease control in Morocco. This isolation process constitutes a first step toward vaccine development.

**Methods:**

Thirteen fecal samples were obtained from calves with a single episode of neonate calf diarrhea at three different dairies and two samples were collected from field during a severe NCD outbreak. Diagnosis of RVA infection was based on fecal immune-chromatographic rapid test and further evaluated for their hemagglutination (HA) activity. RVA isolation was carried out on MA104 cells after inoculates were treated with different concentrations of trypsin TPCK. All RVA isolates were confirmed by LSI VetMAX™ Triplex Ruminant Rotavirus & Coronavirus Real-Time PCR kit. G and P typing were determined by direct sequencing of the VP4 and VP7 amplicons.

**Results:**

RVA isolation was achieved for nine clinical samples following one or two passages (60 %) and was properly depended on HA activity and trypsin treatment of inoculates. The first sign of CPE detected consisted of increased cell granularity, obscure cell boundaries, cell rounding, and eventual degeneration and detachment of cells. At lower TPCK concentration (3–10 μg/inoculum), no changes at the cellular level were observed, while cells activated with 25–30 μg of trypsin/inoculums, they degenerated and trypsin cytotoxicity was enhanced. Appreciable changes in cell’s morphology were detected with optimal trypsin concentration of 15–20 μg trypsin/inoculums. Data from qRT-PCR confirmed that unsuccessful cultivations have No-Ct, and all nine isolates have Ct values ranged between 12.17 and 24.69. Analysis sequencing revealed that field isolates were of G6 P[5] serotype and isolates from the dairy NCD samples were of G10 P[14] serotype.

**Conclusions:**

To our knowledge, this is the first study in Morocco which reports the circulation of G10P[14] in NCD on dairy farms and G6P[5] in the field. Our study constitutes a crucial and a necessary step allowing preventive and veterinary medicine to support RVA disease controls in the country.

## Background

Group A rotaviruses (RVA) are a member of the family *Reoviridae*, genus Rotavirus. RVA infect both animals [18] and humans [5], and cause an acute gastroenteritis (AGE) accompanied by abdominal pain, fever, nausea, and vomiting. The genome of these viruses is composed of 11 segments of dsRNA and is surrounded by three concentric layers of proteins [9]. The outermost layer is formed by two proteins, VP4 and VP7. Dimers of VP4 form spikes that extend from the virus surface and have essential functions in the virus life-cycle, including receptor binding and cell penetration [9].

The diagnosis of RVA was initially based on electron microscopy [10], ELISA [23], RNA electrophoresis [21], nucleic acid hybridization [19], immunofluorescence (IF), the conventional reverse transcription-polymerase chain reaction (RT-PCR) [11], or recently the quantitative real time RT-PCR (qRT-PCR) [20]. These tests while faster, highly sensitive and specific present some limits. They require subsequent virus isolation for the evaluation of agent infectivity. Indeed, virus isolation remains the gold standard test to recover the viral particles from clinical fecal samples, to determine their behavior in tissue culture and to measure their infectivity. In addition, isolation remains the basic assay to provide antigens that can be used as successful immunogenics to raise prominent antibodies which can be useful for the construction and development of a wide variety of immunoassays (rapid immunochromatographic strip test, microneutralization assays (SNT), ELISA, IF or qRT-PCR standards). Thus, the direct cultivation method would be necessary when large numbers of samples need to be analyzed during epidemiological surveys, vaccine trials or when further sensitivity is required. Moreover, efficient and accurate isolation of RVA is of primary importance for pathogenesis, vaccine development and academic research.

In Morocco, no report is available regarding isolation and cultivation of RVA in clinical samples from domestic animals or children with AGE and burden due this infection in animals remain unknown. Hence, this study aims at the isolation and virus characterization of bovine RVA strains from suspected diarrheal clinical cases to support RVA disease control in Morocco. Consequently, this will provide RVA isolates that could be utilized in future vaccine development.

## Results and discussion

Attempts of RVA isolation were conducted with 15 fecal samples from neonate calves submitted to the Laboratory of Biopharma, Rabat, between January and April 2014. A highly permissive and well established fetal rhesus monkey kidney cell line (MA104) was used [2]. Since most RVA strains from bovine are able to agglutinate erythrocytes, these samples were chosen for their ability to agglutinate chicken red blood cells (RBCs) (Table 1) [15, 17], and their reactivity against a highly sensitive and specific immunochromatography test for RVA rapid diagnosis (Rota-Check-1 test -Veda-lab France-).

**Table 1:** Record data from 15 fecal samples collected during 2014, from different geographical regions of Morocco

Samples identification	Calves age (weeks/days)	Regions	Hemagglutination test (HA)	Cell culture passages (P)				Rt-PCR Ct values	Genotypes–Serotypes
				P1	P2	P3	P4		
3∕4	2.2	Laarayche	1/32	+	−	−	−	No Ct	ND
1/13	3.5	Kenitra	1/32	+	−	−	−	No Ct	ND
1/18	3.3	Kenitra	1/64	+	−	−	−	No Ct	ND
2/15	4.4	Moulay bouslham	1/32	+	−	−	−	No Ct	ND
1/25	3.5	Kenitra	1/8	+	−	−	−	No Ct	ND
2/23	3.3	Moulay bouslham	1/64	+	−	−	−	No Ct	ND
3/T	2.5	Khenifra	1/64	−	+	+	+	12.17	G6P[5]
7∕T	2.0	Khenifra	1/8	−	+	+	+	18.52	G6P[5]
3∕25	3.1	Laarayche	1/16	+	+	+	+	23.30	G10P[14]
3∕34	3.2	Laarayche	1/32	+	+	+	+	23.28	G10P[14]
1∕10	3.0	Kenitra	1/16	+	+	+	+	24.69	G10P[14]
2∕20	2.1	Moulay bouslham	1/32	+	+	+	+	20.30	G10P[14]
2∕12	2.1	Moulay bouslham	1/64	+	+	+	+	15.39	G10P[14]
8045	3.2	Kenitra	1/64	+	+	+	+	12.22	G10P[14]
9864	2.4	Laarayche	1/32	+	+	+	+	12.91	G10P[14]

*HA* hemagglutinin titer of the samples, *P* cytopathic effects observed (+) on the first passage (P1) and on P2, P3, P4 respectively on second, third and fourth passages, *RT-PCR* results from qRT-PCR given as Ct values. The Ct is defined as the threshold cycle, *ND* not determined

Prior to this study, we have attempted to recover RVA strains from clinical samples which were subjected to multiple rounds of freeze–thaw (1/13, 1/18, 2/15, 1/25, 2/23 and 3/4). We realized that hemagglutination (HA) of chicken erythrocytes by RVA particles decreased three to eight folds when a sample is freeze–thawed at room temperature and the loss of HA was irreversible after three rounds of freeze–thaw (data not shown). This observation, although reported not to alter the virus morphology [3, 4], is consistent with earlier biological studies suggesting that effective HA of a RVA strain may increase its infectivity and therefore, RVA isolation [15]. Indeed, HA phenotype of RVA is mediated by the VP4 gene which was clearly shown to code for protease enhanced plaque formation in MA104 cells [15]. This VP4 gene, was also shown to be protease-sensitive. It is cleaved to VP5 (60 kDa) and VP8* (28 kDa) in the presence of trypsin, resulting in the conversion of noninfectious rotavirus to an infectious form [7, 8].

**Fig. 1:**
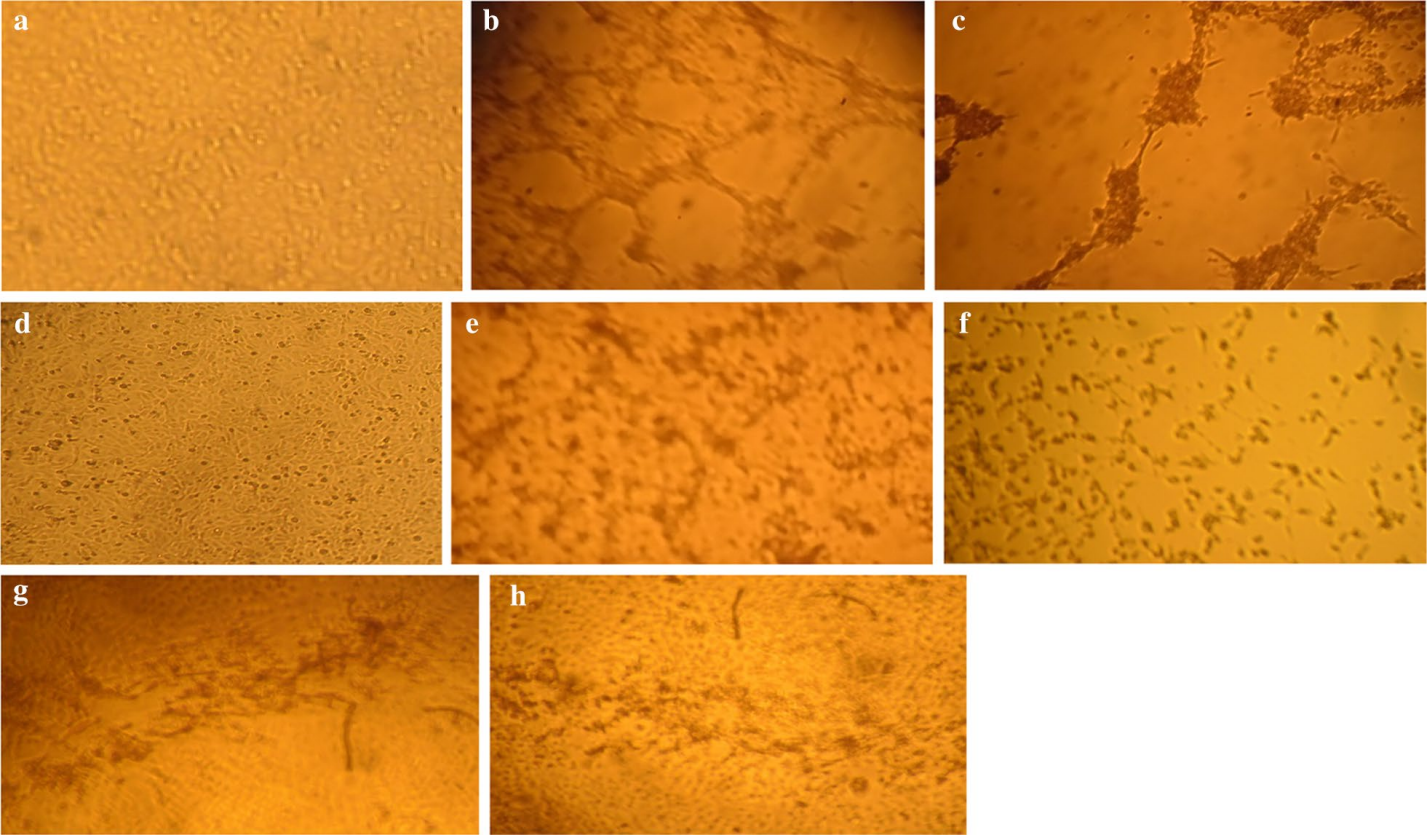
Changes observed at the cellular level after MA104 infection with different bovine RVA strains or controls activated with different concentrations of TPCK-Trypsin (0–30 µg/inoculum). **a** No changes at the cellular level were observed and the shape of the cells was similar to the control cell with 0–10 μg/inoculum. **b**, **c** Cell lysis and degeneration due to trypsin cytotoxicity with 25–30 μg/inoculum. **d** The first sign of cytopathic effect (CPE) with considerable changes in cell’s morphology at 15–20 μg/inoculum. **e**, **f** Cells destruction after 4 days post-infection with 15–20 μg/inoculum. **g**, **h** Cells were apically infected and CPE was optimally observed at 15 µg/inoculum

As the infectivity of RVA is increased by trypsin treatments [6], three bovine samples (3/T, 7/T and 8045) and controls (monolayer cells inoculated with DMEM serum free medium) were exposed to different concentrations of TPCK-Trypsin (3–30 μg/inoculum). The results demonstrate that at lower concentration (i.e., 3–10 μg of trypsin), no changes at the cellular level were observed (Fig. 1a) after six passages and samples were considered negative for RVA isolation. On the contrary, cells activated as well as controls degenerated and trypsin cytotoxicity was enhanced with 25–30 μg of trypsin (Fig. 1b, c). In parallel, when samples were assayed with 15–20 μg of trypsin for 60 min at 37 °C (or 2 h at room temperature) (data not shown), appreciable changes in cell’s morphology were detected 2–3 days post infection (pif) (Fig. 1d). RVA isolation was achieved for nine clinical samples following one or two passages (60 %). CPE was observed during the first passage for samples 3/25, 3/34, 1/10, 2/20, 2/12, 8045 and 9864. The first sign of CPE detected consisted of increased cell granularity, obscure cell boundaries, cell rounding, and eventual degeneration and detachment of cells. This phenomenon was more pronounced when samples were activated with 20 μg of TPCK. CPE were consistently observed thereafter and the cells became completely destroyed after 4 days pif (Fig. 1e, f). For two samples (3/T and 7/T), the CPE effect was observed in the second passage on day 2 pif. Therefore, we consider that 15 μg/inoculum of TPCK was the optimal concentration for bovine RVA isolation (Fig. 1g, h).

On the other hand, the recovery of RVA from samples 1/13, 1/18, 2/15, 1/25, 2/23 and 3/4 was not achieved. This is explained by the fact that infected cells were not washed sufficiently to remove FCS which inhibits trypsin and not to their initial HA titers. Indeed, RVA strains from 2/12 and 7/T having the same HA titers (1/64 and 1/8 respectively) as 2/23 and 1/25 samples were successfully isolated when infected cells were adequately washed (Table 1). Also, other factors were interfering with successful isolation such the infectious viral titer in the inoculums, inadequate storage of samples and multiple rounds of freeze-thaw which decline their HA titers.

As the presence of trypsin in the culture medium during multiple cycles of virus replication was demonstrated to enhance the infectivity of rotavirus between 10- and 1000-fold [12], we could demonstrate that a concentration of 0.5–1 μg/ml in the serum-free DMEM medium was optimal for virus penetration. However, a concentration of 2 μg/ml resulted in the lysis of cell controls. Whether trypsin concentration (15 or 20 μg/inoculum) on viral activation or penetration (0.5–1 μg/ml) affect the viral titer or not was not checked.

The role of proteolytic enzyme (i.e., trypsin) in the penetration of RVA is well demonstrated [6, 16] and our results corroborate the previous ones showing that this enzyme is indispensable for the entry of the virus into a host cell [1]. Data from qRT-PCR confirmed that 3/T, 7/T, 3/25, 3/34, 1/10, 2/20, 2/12, 8045 and 9864 isolates were of group A rotavirus with Ct values ranging between 12.17 and 24.69. However, no RNA loads (No Ct in Table 1) were detected for 1/13, 1/18, 2/15, 1/25, 2/23 and 3/4. G and P typing were performed for all strains, and analysis sequencing revealed that two isolates (3/T and 7/T) were of G6P[5] serotype and seven (3/25, 3/34, 1/10, 2/20, 2/12, 8045, 9864) were of G10P[14] serotype. (Table 1).

## Conclusion

To our knowledge, this is the first study in Morocco which reports the circulation of G10 P[14] in dairy calves and G6P[5] in the field. Our study constitutes a crucial and a necessary step allowing preventive and veterinary medicine to support RVA disease controls in the country. RVA could be isolated from 60 % of clinical fecal samples and the efficient isolation procedure depends on four major factors:

- The use of a sensitive and permissible cell line, such as MA104.
- The use of an optimal trypsin concentration both for viral activation (10-15 µg/inoculum) and penetration and propagation (0.5–1 µg/ml).
- An adequate washing of cells before viral adsorption.
- An adequate storage of viral particles after collection.

## Methods

### Bovine fecal samples

Thirteen fecal samples were obtained from calves with a single episode of neonate calf diarrhea at three different dairies and two samples were collected from field during a severe NCD outbreak. All samples were stored at −80 °C in Virology Laboratory of Biopharma, their records data are summarized in Table 1. Diagnosis of RVA infection was based on positive fecal immune-chromatographic rapid test (Rota-Check-1 test -Veda-lab France-). All isolated were evaluated for their hemagglutination (HA) activity using red blood cells as described by the microtiter method [13]. Hemagglutination titers were determined by serial dilutions of 50 µl of antigen in Phosphate buffered saline (PBS) (CAT No. 211-410-QK, Wisent Bioproducts). These were mixed with 50 µl of chicken’s erythrocyte suspension (0.1 %). The mixtures were incubated for 30 min at room temperature. The titer was expressed as the reciprocal of the highest dilution of antigen showing complete hemagglutination.

### Ethical approval

All fecal samples were collected at three different dairies after authorization and in accordance with the Vet responsible, Dr. M. Chadli.

### MA104 cell line growth and maintenance

Permanent cultures of fetal rhesus monkey kidney cell line (MA104) were used. These cells were kindly provided by Prof. P. Pothier from the Centre National des virus Entériques (CNR), Dijon, France. They were grown in 80 cm^2^ Nunc flasks in Dulbecco’s modified Eagle’s medium (DMEM) (CAT No. 219-015-QK, Wisent Bioproducts) containing 10 % fecal calf serum (FCS) (CAT No. 080150, Wisent Bioproducts) with 100 U/ml penicillin G and 100 μg/ml streptomycin. Cells were incubated at 37 °C until confluence.

For viral infection, cells were used in a 25 cm^2^ flasks in order to have cell confluences (24 h).

### Rotavirus isolation

For each sample, 2 ml of feces were put into tube containing 6 ml of PBS with glass beads. Samples were then clarified by centrifugation at 2500 rpm for 30 min. The supernatants from the homogenates were filtered through 0.45 µm membrane (Sigma) and stored at −80 °C until use.

Only three fecal samples were subjected to different treatments of trypsin. They were (3/T, 7/T and 8045). 500 µl of each bovine filtrate was treated with different increasing final concentrations (3, 5, 10, 15, 20, 25, 30 µg per inoculums of TPCK-Trypsin) (CAT No. 9002-93-1, Sigma) for 1 h in a 37 °C water bath after a brief vortex to mix. In parallel, for each trypsin concentration, 0.5 ml of DMEM serum-free medium was prepared and constitutes the mocked-controls. For efficient infection of the cells, growth medium was removed from MA104 cell monolayers and washed one time with 4 ml of pre-warmed serum-free DMEM medium. This step is essential; the presence of serum inhibits RVA activation [2].

Immediately after activation, samples (or controls) were inoculated into confluent monolayer of MA104 cells and incubated at 37 °C, with 5 % CO_2_ for 60 min to allow adsorption. Subsequent to viral adsorption, cells were observed under the microscope to detect any cell degeneration due to trypsin and had been washed once using 8 ml of pre-warmed, serum-free DMEM for each flask. The wash is removed and 8 ml of pre-warmed serum-free medium is added with 1 μg/ml of TPCK. Then, the infected-MA104 cell flasks and controls were checked daily for 3–6 days or until a cytopathic effect (CPE) appeared.

The remaining samples were treated with 15 µg of trypsin per inoculum and preceded as described above.

For serial passages, infected cells were freeze-thawed and inoculated onto fresh confluent monolayer cells for another passage after trypsin treatment as described above. Cells not showing CPE after six passages were considered negative for RVA isolation.

### RVA identification and genotyping

Nucleic acid extraction was performed by MagMAX kit following the manufacturers’ instructions (Life technologies). All RVA isolates were confirmed by LSI VetMAX™ Triplex Ruminant Rotavirus & Coronavirus Real-Time PCR kit (Life technologies, France). G and P typing assays for positive samples were conducted by RT-PCRs using Qiagen one step kit with the VP7 and VP4 primers described previously [14, 22].

Genotyping of VP4 and VP7 was performed by direct sequencing the VP4 or VP7-derived first round amplicons with the same set of primers as for amplification. Amplicons were purified using the ExoSAP-IT (Affymetrix) preceding to sequencing with the BigDye Terminator v3.1 (Applied Biosystems). Sequences were determined using ABI 3130xl automate (Applied biosystems).

### Nucleotide sequence accession numbers

The nucleotide sequences data have been submitted to GenBank under the following accession numbers: KU729665, KU729669, KU729666, KU729670, KU729667, KU729671, KU729668 and KU729672.

## Abbreviations

AGE: acute gastroenteritis
CPE: cytopathic effect
DMEM: Dulbecco’s modified Eagle’s medium
dsRNA: double-stranded ribonucleic acid
ELISA: enzyme-linked immunosorbent assay
FCS: fecal calf serum
HA: hemagglutination
IF: immunofluorescence
NCD: neonatal calve diarrhea
PBS: phosphate buffered saline
qRT-PCR: quantitative real time reverse transcription-polymerase chain reaction
RNA: ribonucleic acid
RT-PCR: reverse transcription-polymerase chain reaction
RVA: group A rotaviruses
SNT: microneutralization assays
